# Exogenous abscisic acid prolongs the dormancy of recalcitrant seed of *Panax notoginseng*

**DOI:** 10.3389/fpls.2023.1054736

**Published:** 2023-02-14

**Authors:** Qing-Yan Wang, Ling Yang, Na Ge, Jin-Shan Jia, Rong-Mei Huang, Cui Chen, Zhen-Gui Meng, Long-Gen Li, Jun-Wen Chen

**Affiliations:** ^1^ College of Agronomy and Biotechnology, Yunnan Agricultural University, Kunming, China; ^2^ Key Laboratory of Medicinal Plant Biology of Yunnan Province, Yunnan Agricultural University, Kunming, China; ^3^ National and Local Joint Engineering Research Center on Germplasm Innovation and Utilization of Chinese Medicinal Materials in Southwestern China, Yunnan Agricultural University, Kunming, China

**Keywords:** recalcitrant seed, dormancy, abscisic acid, after-ripening process, *Panax notoginseng*

## Abstract

The seeds of *Panax notoginseng* (Burk.) F. H. Chen are typically characterized by their recalcitrance and after-ripening process and exhibit a high water content at harvest as well as a high susceptibility to dehydration. Storage difficulty and the low germination of recalcitrant seeds of *P. notoginseng* are known to cause an obstacle to agricultural production. In this study, the ratio of embryo to endosperm (Em/En) in abscisic acid (ABA) treatments (1 mg·l^−1^ and 10 mg·l^−1^, LA and HA) was 53.64% and 52.34%, respectively, which were lower than those in control check (CK) (61.98%) at 30 days of the after-ripening process (DAR). A total of 83.67% of seeds germinated in the CK, 49% of seeds germinated in the LA treatment, and 37.33% of seeds germinated in the HA treatment at 60 DAR. The ABA, gibberellin (GA), and auxin (IAA) levels were increased in the HA treatment at 0 DAR, while the jasmonic acid (JA) levels were decreased. ABA, IAA, and JA were increased, but GA was decreased with HA treatment at 30 DAR. A total of 4,742, 16,531, and 890 differentially expressed genes (DEGs) were identified between the HA-treated and CK groups, respectively, along with obvious enrichment in the ABA-regulated plant hormone pathway and the mitogen-activated protein kinase (MAPK) signaling pathway. The expression of *pyracbactin resistance-like* (*PYL*) and *SNF1-related protein kinase subfamily 2* (*SnRK2s*) increased in the ABA-treated groups, whereas the expression of *type 2C protein phosphatase* (*PP2C*) decreased, both of which are related to the ABA signaling pathway. As a result of the changes in expression of these genes, increased ABA signaling and suppressed GA signaling could inhibit the growth of the embryo and the expansion of developmental space. Furthermore, our results demonstrated that MAPK signaling cascades might be involved in the amplification of hormone signaling. Meanwhile, our study uncovered that the exogenous hormone ABA could inhibit embryonic development, promote dormancy, and delay germination in recalcitrant seeds. These findings reveal the critical role of ABA in regulating the dormancy of recalcitrant seeds, and thereby provide a new insight into recalcitrant seeds in agricultural production and storage.

## Introduction

The seed is divided into orthodox, intermediate, and recalcitrant types according to its storage behavior (Roberts, 1973). Orthodox seeds experience a dehydrating process at maturation and might be stored with a low water content (Matilla and Matilla-Vázquez, 2008). Correspondingly, recalcitrant seeds maintain a high-water content when they mature and fall off, thus making them sensitive to dehydration (Roberts, 1973; Smith and Berjak, 1995; Berjak and Pammenter, 2001). The viability is significantly reduced, and the germinability is obviously low, when the recalcitrant seeds of *Acer saccharinum* are dehydrated to be about 30% water content (Pukacka and Ratajczak, 2006; Kalemba and Ratajczak, 2018), and when the recalcitrant seeds of *Castanea sativa* are dehydrated to be below 17.4% water content (Costa et al., 2018). The recalcitrant *Richilia dregeana* seeds could maintain viability for several weeks at 16°C at the harvested water content, but they only exhibit 4% viability after 8 days of storage at the axis water content of 1.68 g·g^−1^ dry mass (Drew et al., 2000). The recalcitrant *Araucaria angustifolia* seeds show a complete loss of embryo viability after storage at 48% water content for 26 months (Gasparin et al., 2020). The dehydration sensitivity of recalcitrant seeds makes them impossible for long-term storage.

Seed dormancy is a strategy for plants to adapt to adverse environments during long-term phylogenies (Gubler et al., 2005; Song et al., 2008). Most recalcitrant seeds usually germinate immediately after they are shed from mother plants (Pritchard, 2004; Graeber et al., 2012), but some recalcitrant seeds show a markedly dormant characteristic (Liu et al., 2005). Morphophysiological dormancy (MPD) is one of the types of seed dormancy, and it is generally caused by incompletely developed embryos (Willis et al., 2014). The embryo of *Paeonia lactiflora* seeds with MPD dormancy is incompletely developed at harvest and thus has an after-ripening process of 75 days (Lu et al., 2022). After-ripening is a necessary process for MPD seeds before germination (Baskin and Baskin, 2004; Zhang and Shen, 2006; Chen et al., 2008; Yu et al., 2012). Abscisic acid (ABA) is continuously decreased and gibberellin (GA) is significantly increased in the MPD seeds of *Musella lasiocarpa* during the after-ripening process (Tang, 2014). The increase in endogenous GA and the changed rate of GA/ABA could promote the embryonic development of *Paris polyphylla* var. *Yunnansensis* seeds during the after-ripening process and consequently release dormancy (Pu et al., 2016). *Panax quinquefolium* seeds are characterized by MPD and a long period of dormancy, and exogenous GA treatment could promote them to complete the physiological after-ripening process (Zhao et al., 2000). However, it is not well investigated whether or not exogenous hormones play an important role in regulating the dormancy of recalcitrant seeds at the transcriptional level.

ABA is a critical positive regulator to maintain seed dormancy (Hilhorst, 1995; Kermode, 2005; Kucera et al., 2005). The germination of ABA-deficient mutant seeds is earlier than that of wild-type seeds (Frey et al., 2011), whereas dormancy is enhanced in transgenic lines of ABA-overaccumulated seeds (Qin and Zeevaart, 2002). ABA is highly accumulated in ABA catabolism mutants, which enhances the dormancy of *Arabidopsis thaliana* seeds (Matakiadis et al., 2009). The *de novo* synthesis of GA is necessary to release dormancy and is antagonistic to ABA in regulating seed dormancy (Shuai et al., 2017). GA-deficient mutants *ga1* and *ga2* exhibit enhanced dormancy and inhibited germination (Lee et al., 2002; Shu et al., 2013). The regulation of ABA/GA rate is associated with the metabolic transition required for dormancy release and germination (Yamaguchi, 2008; Nambara et al., 2010), and the maintenance of dormancy depends on a high rate of ABA/GA. The increased GA biosynthesis and the increased ABA degradation result in a low rate of ABA/GA, consequently releasing dormancy (Ali-Rachedi et al., 2004; Cadman et al., 2006). In addition, the application of exogenous auxin (IAA) prevents pre-harvest sprouting in wheat (*Triticum aestivum*) seeds (Ramaih et al., 2003). It has been demonstrated that the synergistic effect of jasmonic acid (JA) and ABA inhibits seed germination in several species, including *Brassica napus*, *Linum usitatissimum*, and *A. thaliana* (Staswick et al., 1992; Ellis and Turner, 2002). Nevertheless, relatively little effort has been made to investigate the effect of the exogenous hormone ABA on the regulation of recalcitrant seed dormancy.

The PYR/PYL/RCAR-PP2C-SnRKs signaling cascade is a major pathway for ABA to work (Cutler et al., 2010; Hubbard et al., 2010). ABA receptors *pyracbactin resistance* (*PYR*), *pyracbactin resistance-like* (*PYL*), and the regulatory component of ABA receptors (RCARs) could sense the ABA signal and inhibit the type 2C protein phosphatases (PP2Cs) in *A. thaliana* (Park et al., 2009; Tischer et al., 2017). The *SNF1-related protein kinase subfamily 2* (*SnRK2*) positively regulates ABA signaling, as evidenced by the fact that the *snrk2.2 snrk2.3 snrk2.6* triple mutant is insensitive to ABA (Fujii and Zhu, 2009; Nakashima et al., 2009). ABA signaling is not the only factor in regulating seed dormancy (Carrera et al., 2008). For example, the seeds of *gid1a gid1b gid1c* multiple mutants are dormant and unable to germinate in the absence of exogenous GA (Griffiths et al., 2006; Iuchi et al., 2007; Voegele et al., 2011), and it has been proven that MAPK signaling cascades are involved in ABA signal transduction and in regulating seed germination (Xing et al., 2008; Jammes et al., 2009). However, it is still not well understood about the role of endogenous ABA signaling in responding to exogenous hormone in recalcitrant seeds during the after-ripening process.

*Panax notoginseng* (Burk.) F. H. Chen belongs to the genus *Panax* in the Araliaceae family. The seeds are believed to be typically characterized by recalcitrance and the after-ripening process, having high water content at harvest and being sensitive to dehydration (Cui et al., 1993; Duan et al., 2014). The seed embryos of *P. notoginseng* belong to the type of MPD (Duan et al., 2010a; Duan et al., 2010b). The 30 days of after-ripening process (DAR) is a critical point for the release of the dormancy of *P. notoginseng* seeds, and it needs 45–60 days of lamination to complete the after-ripening of the embryos before germination (Yang et al., 2018; Yang et al., 2019; Ge et al., 2021). Our previous study has demonstrated that endogenous ABA shows a decreasing trend, while GA first increases and then decreases in *P. notoginseng* seeds during the after-ripening process (Yang et al., 2018), and some candidate genes related to seed dormancy have been obtained in *P. notoginseng*, including *ABSCISIC ACID-INSENSITIVE 5* (*ABI5*), *XANTHOXIN DEHYDROGENASE* (*ABA2*), *PROTEIN PHOSPHATASE 2C* (*PP2C*), *DELLA PROTEIN GA-INSENSITIVE* (*GAI*), and *GAINSENSITIVE DWARF 2* (*GID2*) (Yang et al., 2019). Our work has also shown that exogenous ABA treatment disturbs the balance of endogenous hormones, prolonging the after-ripening process in *P. notoginseng* seeds (Ge et al., 2021). However, it is still unclear whether exogenous ABA influences the dormant regulation of recalcitrant *P. notoginseng* seeds at the transcriptional level. In this study, the morphology of embryos, the endogenous hormones, and the differentially expressed genes (DEGs) were determined in control check (CK) and HA-treated *P. notoginseng* seeds. The results would provide a new insight into the mechanism of dormant regulation in recalcitrant seeds with after-ripening characteristics.

## Materials and methods

### Plant materials

In November, the mature and plump seeds were selected from the 3-year-old *P. notoginseng* that was planted at the experimental farm of Miaoxiang Sanqi Technology Company in Wenshan City, Yunnan Province, China. After harvesting, the red peels of seeds were removed, and then disinfected with 5% CuSO_4_ for 30 min, rinsed with double distilled water (ddH_2_O) twice, and dried indoors. The seeds were soaked in ABA hormone solution for 24 h, and their mass concentrations are 1 mg·l^−1^ and 10 mg· l^−1^ (LA and HA). The ratio is 1:3–4 between the seeds and the soaking solution, and the CK was treated with distilled water. Subsequently, the seeds were mixed with wet sand after high-temperature disinfection at a volume ratio of 1:5, and the humidity of the river sand is about 25%. The seeds were placed in well-ventilated, fully meshed baskets in a sand-laminated chamber at 15 ± 5°C and kept away from light for 50–60 days to accomplish the after-ripening process. Each treatment was designed with three replicates. At each sampling point (0, 30, and 50 DAR), the samples of the CK and ABA-treated groups were selected for further assays.

### Measurement of morphological and physiological indicators

Seeds were selected to observe seed development at five time points: 0, 15, 30, 45, and 60 DAR. The seeds of *P. notoginseng* were cut along the embryo lengthwise and placed under a stereoscopic microscope (SteREO Discovery V.20, Zeiss, Germany, RRID : SCR_016980) equipped with a digital camera to measure the lengths of embryo (Em) and endosperm (En), and thus the rate of embryo and endosperm (Em/En) was calculated. The measurement was repeated 20 times independently, and three biological replicates were performed. At 45 DAR, the seeds were placed in seeding trays (100 seeds per tray) containing wet sand and incubated at 20°C in an incubator. The number of germinating seeds was recorded and counted until 60 DAR. The contents of endogenous hormones were determined at 0, 30, and 50 DAR by using HPLC-MS (high-performance liquid chromatography-tandem mass spectrometry), including ABA, GA, IAA, and JA in the CK and ABA-treated *P. notoginseng* seeds (Pan et al., 2010).

### Total RNA extraction and transcriptome analysis

The TaKaRa MiniBEST Plant RNA Extraction Kit was used to extract total RNA from samples. The quality of RNA samples was tested by using 1% agarose electrophoresis and an Agilent 2100 Bioanalyzer (Agilent Technologies, Palo Alto, CA, USA, RRID : SCR_013575). Sequencing libraries were obtained using the NEBNext^®^ UltraTM RNA Library Prep Kit for Illumina^®^ (NEB, USA, RRID : SCR_010233), and they were sequenced on an Illumina platform (NEB, USA, RRID : SCR_010233) by the Novegene Technology Company (Beijing, China). The clean reads were obtained by removing low-quality and adapter-containing reads and then compared to the *P. notogensing* reference genome (Fan et al., 2020) using HISAT2 v2.0.5 (RRID : SCR_015530). The transcript abundances of genes were estimated by fragments per kilobase of transcript per million fragments mapped (FPKM). The distribution of log2(FPKM + 1) showed relatively high gene expression (**Figure S1**). Correlation analysis was performed to examine the repeatability of the experiment, and the reliability of pairwise correlation of all samples was assessed using the Pearson Correlation Coefficient (R) (**Figure S2**). All R^2^ values were close to 1 between the three biological replicates of the six samples in this experiment, indicating that the sequencing quality of the transcriptome was satisfactory.

In this study, we used the DESeq2 R package (1.16.1, RRID : SCR_015687) to compare the treated group with the CK group. According to the method of Benjamini and Hochberg (1995), genes were designated as having differential expression at *P*-values < 0.05. The clusterProfiler R package (RRID : SCR_016884) was used for GO and KEGG analyses.

### Validation of gene expression

The validation of gene expression was determined by quantitative real-time PCR (qRT-PCR). Primers for qRT-PCR were designed using the Primer3 website (https://primer3.ut.ee/ , RRID : SCR_003139) and synthesized by Tsingke Biotech Co., Ltd. (Yunnan, China). All primers are shown in **Table S2**. The RNA was synthesized into single-strand cDNA by the Prime Script RT reagent kit after extracting from each sample (Takara Bio, Kyoto, Japan, RRID : SCR_021372). The QuantStudio12 K Flex System (Thermo Fisher Scientific, RRID : SCR_008452) was used to perform PCR with three technical replicates. The *GLYCERALDEHYDE-3-PHOSPHATE DEHYDRO GENASE* (*GAPDH*) was selected as the internal reference for *P. notoginseng* seeds. The relative expression of the candidate genes was calculated using the 2^−ΔΔCt^ method (Livak and Schmittgen, 2001).

### Statistical analysis of data

GraphPad Prism 8 (RRID : SCR_002798) and Adobe Photoshop CS6 (RRID : SCR_014199) were used for mapping, and Adobe Illustrator Artwork 21.0 was used for a combination. Statistics were analyzed using SPSS 26.0 (RRID : SCR_002865) by one-way ANOVA and Duncan’s multiple comparison method for a significant difference analysis (*P <* 0.05), and the variables are displayed as the mean ± SD (*n* = 3).

## Results

### Effects of ABA treatment on physiological indexes of *P. notoginseng* seeds

**Figure 1:**
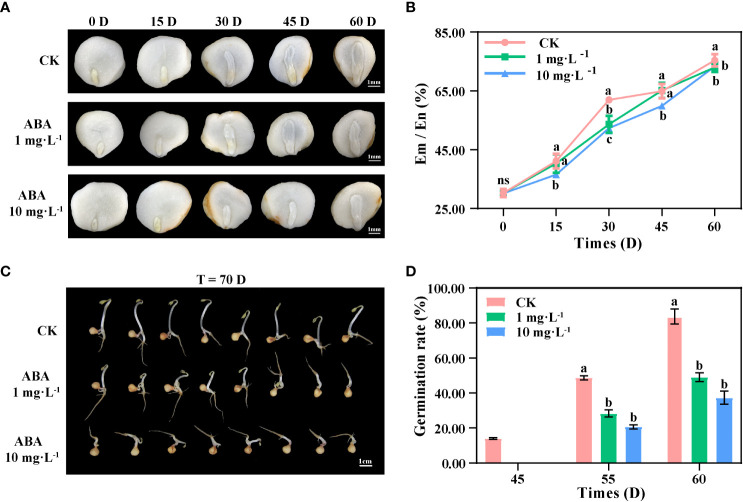
Exogenous ABA inhibits the development of seed embryos and delays seed germination in *Panax notoginsen*g during the after-ripening process. **(A)** The morphological development of *P. notoginseng* seed embryos. **(B)** Changes in the rate of Endosperm to Embryo (Em/En) of *P. notoginseng* seeds. **(C)** Appearance and morphology of *P. notoginseng* seeds after germination (*T* = 70 DAR (days during after-ripening)). **(D)** Changes in the rate of germination of *P. notoginseng* seeds. Values presented are the means ± SE (*n* = 3). Different letters indicate significant differences among treatments in the same period using Duncan’s test (*P <* 0.05).

In this study, *P. notoginseng* seeds were cultivated in land stratification during the after-ripening process for observing the morphological development process. As shown in **Figures 1A, B**, the development of seed embryos was significantly inhibited during 0–30 DAR. In the LA-treated seeds and HA-treated seeds, the rates of Em/En at 30 DAR were 53.64% and 52.34%, being significantly lower than those in the CK (61.98%), whereas at 45 DAR, the rates of Em/En were 64.88% (CK), 65.16% (LA), and 59.92% (HA), respectively. It indicated that the development of seed embryos was delayed for at least 15 days under the HA treatment at the early stage of the after-ripening process. The development of seed embryos seemed to be accelerated with the ABA treatments after 30 DAR; the rates of Em/En in the CK, LA-treated seeds, and HA-treated seeds reached about 75% at 60 DAR (**Figures 1A, B**), indicating that the exogenous ABA had an inhibitory effect on the embryonic development of *P. notoginseng* seeds at the early after-ripening stage and that the inhibition of embryonic development was deepened by the increased ABA concentration (**Figures 1A, B**).

At 45 DAR, 14% of the CK seeds germinated, while no seeds germinated in the LA-treated groups or the HA-treated groups (**Figure 1D**). At 55 DAR, the germination rate of LA-treated seeds and HA-treated seeds was 28.33% and 20.67%, being significantly lower than that of the CK (48.67%). At 60 DAR, the germination rates of the CK, LA-treated seeds, and HA-treated seeds were 83.67%, 49%, and 37.33%, respectively (**Figures 1C, D**). Our results showed that the germination of LA-treated seeds was delayed for about 5 days, while that of HA-treated seeds was delayed for more than 10 days.

### Effects of ABA treatment on endogenous hormones of *P. notoginseng* seeds

As shown in **Table 1**, the endogenous hormone ABA was extensively accumulated at 0 DAR and then decreased at 30 DAR and 50 DAR; however, the ABA content was increased under ABA treatment. Exogenous ABA increased GA content at 0 DAR, whereas endogenous GA accumulation was inhibited in the HA-treated seeds at 30 DAR and 50 DAR (**Table 1**). During the after-ripening process, the IAA content was gradually decreased in the CK and HA-treated seeds, but the IAA content was higher in the HA-treated seeds than the ones in the CK at 0 and 30 DAR (**Table 1**). The JA content was increased in both the CK and HA-treated seeds during the after-ripening process; however, in the HA-treated seeds, the JA content was higher than that in CK seeds at 30 and 50 DAR (**Table 1**). Specifically, JA content was decreased in HA-treated seeds at 0 DAR (**Table 1**). Furthermore, the reduction in GA and the increase in ABA resulted in a low rate of ABA/GA under the ABA treatment when compared with the CK (**Tables 1**, **2**).

**Table 1:** Changes of endogenous hormones in the control (CK) and10 mg·l^−1^ ABA-treated (HA) *P. notoginseng* seeds during the after-ripening process.

Endogenous hormones	Time (DAR)	Treatments	
		CK (ng·g^−1^)	10 mg·l^−1^ ABA (ng·g^−1^)
ABA	0 D	0.25 ± 0.06 b	2.83 ± 0.92 a
	30 D	0.15 ± 0.02 a	0.16 ± 0.16 a
	50 D	0.12 ± 0.03 a	0.12 ± 0.01 a
GA	0 D	0.43 ± 0.10 a	0.77 ± 0.06 a
	30 D	0.20 ± 0.20 a	0.17 ± 0.17 a
	50 D	0.29 ± 0.10 a	0.15 ± 0.03 a
IAA	0 D	20.47 ± 1.90 a	23.80 ± 3.52 a
	30 D	12.70 ± 1.35 a	13.91 ± 1.05 a
	50 D	7.11 ± 2.93 a	6.70 ± 0.29 a
JA	0 D	0.18 ± 0.03 a	0.17 ± 0.01 a
	30 D	0.23 ± 0.01 b	0.64 ± 0.08 a
	50 D	0.96 ± 0.22 a	1.25 ± 0.18 a

Values presented are the means ± SE (*n* = 3). Different letters indicate significant differences among treatments in the same period using Duncan’s test (P < 0.05).

**Table 2:** Changes in the ratio of endogenous hormones ABA to GA (ABA/GA) in *P. notoginseng* seeds during the after-ripening process. .

Time (DAR)	Treatments		
	CK	1 mg·l^−1^ ABA	10 mg·l^−1^ ABA
0 D	0.58 ± 0.05 b	0.09 ± 0.01 b	3.67± 1.16 a
30 D	0.83 ± 0.18 a	0.82 ± 0.02 a	0.94 ± 0.20 a
50 D	0.62 ± 0.33 a	0.82 ± 0.27 a	0.81 ± 0.15 a

Values presented are the means ± SE (*n* = 3). Different letters indicate significant differences among treatments in the same period using Duncan’s test (P < 0.05).

### RNA-seq and gene annotation of *P. notoginseng* seeds

Based on the previous morphophysiological characteristics, samples of CK- and HA-treated seeds were collected from three time points (0, 30, and 50 DAR) and then were selected to investigate the regulatory pathways at the transcriptional level. Eighteen cDNA libraries (CK_0_1, CK_0_2, CK_0_3, CK_30_1, CK_30_2, CK_30_3, CK_50_1, CK_50_2, CK_50_3, HA_0_1, HA_0_2, HA_0_3, HA_30_1, HA_30_2, HA_30_3, and HA_50_1, HA_50_2, and HA_50_3) were sequenced to identify gene expression information of *P. notoginseng* seeds using Illumina HiSeq. The Q30 of eighteen samples was about 93%, and the base content was uniform (**Table S1**). The clean reads were compared quickly and accurately with the genome of *P. notoginseng* (Fan et al., 2020). In all samples, mapped reads were above 90%, unique mapped reads were above 86%, and multiple mapped reads were below 5.2% (**Table 3**).

**Table 3:** Comparative genome statistics of samples.

Sample	Total reads	Mapped reads	Unique mapped reads	Multiple mapped reads
CK_0_1	46815702	42910019(91.66%)	40899591(87.36%)	2010428(4.29%)
CK_0_2	46919124	42880938(91.39%)	40775136(86.91%)	2105802(4.49%)
CK_0_3	45916162	42104871(91.7%)	40017119(87.15%)	2087752(4.55%)
CK_30_1	46770276	43229185(92.43%)	41469671(88.67%)	1759514(3.76%)
CK_30_2	46087118	42530822(92.28%)	40909769(88.77%)	1621053(3.52%)
CK_30_3	50775950	46563721(91.7%)	44146740(86.94%)	2416981(4.76%)
CK_50_1	46450880	41971359(90.36%)	39989166(86.09%)	1982193(4.27%)
CK_50_2	44037090	40404986(91.75%)	38490509(87.4%)	1914477(4.35%)
CK_50_3	49358510	45429117(92.04%)	43281381(87.69%)	2147736(4.35%)
HA_0_1	46371594	42423231(91.49%)	40335559(86.98%)	2087672(4.5%)
HA_0_2	49958870	45764395(91.6%)	43212512(86.5%)	2551883(5.11%)
HA_0_3	46123694	42407335(91.94%)	40478554(87.76%)	1928781(4.18%)
HA_30_1	45367036	41508211(91.49%)	39549766(87.18%)	1958445(4.32%)
HA_30_2	44814232	41079675(91.67%)	39150450(87.36%)	1929225(4.3%)
HA_30_3	45870888	41875152(91.29%)	39899776(86.98%)	1975376(4.31%)
HA_50_1	44730048	40897348(91.43%)	39038282(87.28%)	1859066(4.16%)
HA_50_2	46684002	42801021(91.68%)	40931415(87.68%)	1869606(4.0%)
HA_50_3	45870028	41942451(91.44%)	40168285(87.57%)	1774166(3.87%)

### DEGs functional annotation and comparative analysis of *P. notoginseng* seeds

**Figure 2:**
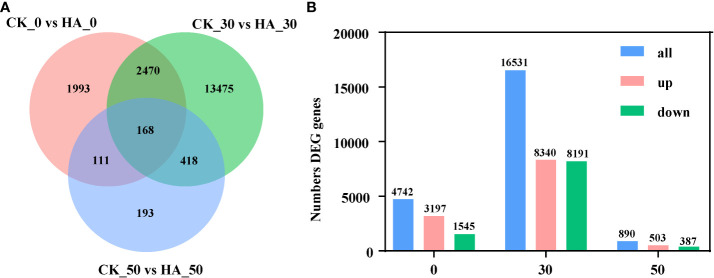
Statistical analysis of differentially expressed genes (DEGs) in *P. notoginseng* seeds during the after-ripening process. **(A)** Venn diagram of DEGs in the control check (CK) and 10 mg·l^−1^ ABA-treated (HA-treated) *P. notoginseng* seeds. **(B)** Histogram of number DEG genes.

**Figure 3:**
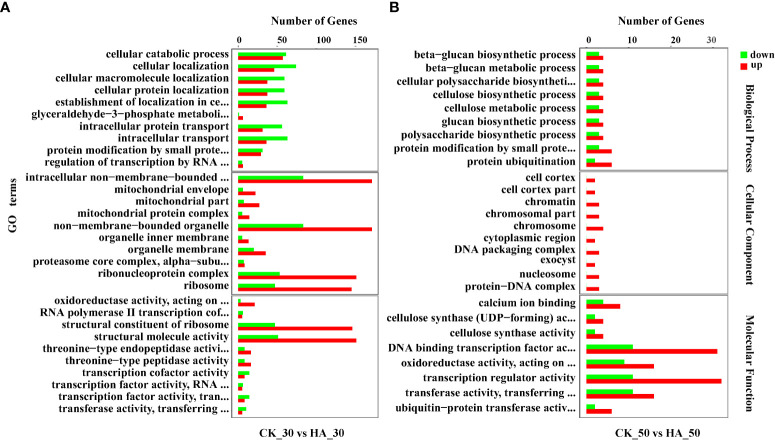
GO functional annotation of differentially expressed genes (DEGs) in the CK and HA-treated *P. notoginseng* seeds. **(A)** Top 30 enriched GO terms of DEGs between CK_30 *vs* HA_30. **(B)** Top 30 enriched GO terms of DEGs between CK_50 *vs* HA_50. The Y-axis on the left represents GO terms, including biological process, cellular component, and molecular function, the X-axis indicates the gene number of each term. Upregulated genes are shown in a red bar, and downregulated genes are shown in a green bar.

A total of 4,742, 16,531, and 890 DEGs were identified in pairwise comparisons of CK_0 *vs* HA_0, CK_30 *vs* HA_30, and CK_50 *vs* HA, respectively (**Figure 2A**). At 0 DAR, 3,197v DEGs were upregulated and 1,545 DEGs were downregulated in HA-treated seeds as compared with CK (**Figure 2B**). At 30 DAR, 8,340 DEGs were upregulated and 8,191 DEGs were downregulated in HA-treated seeds compared with CK (**Figure 2B**). At 50 DAR, 503 DEGs were upregulated and 387 DEGs were downregulated in HA-treated seeds (**Figure 2B**). The functions of DEGs were analyzed using GO annotation. Most of the DEGs were enriched in molecular function (MF) and biological process (BP) in the comparisons at CK_0 vs HA_0, CK_50 vs HA_50. The upregulated DEGs were mainly enriched in cellular component (CC), MF, and the downregulated DEGs were mainly enriched in BP and CC (**Figure 3**; **Figure S3**). In CC classifications, it has been observed that the DEGs were enriched in organelles and organelle parts, and cellular processes, metabolic processes, and stress response cell parts were more active in BP classifications, while binding, catalysis, and transcription activity were more abundant in the MF classifications.

**Figure 4:**
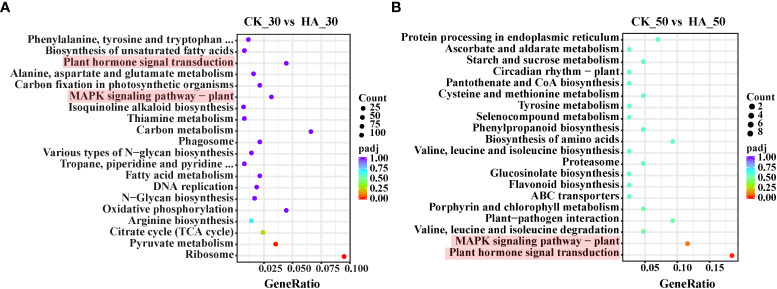
KEGG enrichment analysis of differentially expressed genes (DEGs) in the CK and HA-treated *P. notoginseng* seeds during the after-ripening process. **(A)** Top 20 enriched KEGG pathways of upregulated genes between CK_30 *vs* HA_30. **(B)** Top 20 enriched KEGG pathways of upregulated genes between CK_50 *vs* HA_50. The Y-axis on the left represents KEGG pathways, and the X-axis indicates the “gene ratio” represented by the ratio of upregulated gene numbers to total annotated gene numbers for each pathway. Low padj values are shown in the red circle, and high padj values are shown in the purple circle. The area of a circle represents DEG numbers.

KEGG analysis revealed that the 442 upregulated differential genes were enriched in 97 pathways between the CK_0 and HA_0 groups, including plant–pathogen interaction, protein processing in the endoplasmic reticulum, the MAPK signaling pathway-plant, plant hormone signal transduction, and the biosynthesis of cofactors (**Figure S4A**). The 319 downregulated differential genes were enriched in 89 pathways, mainly the metabolism of amino acids and sugars, the citrate cycle (TCA cycle), the biosynthesis of amino acids and pantothenate, and CoA biosynthesis (**Figure S4B**). In the CK_30 and HA_30 groups, 1,732 upregulated differential genes were enriched in 115 pathways, including biosynthesis and metabolism of amino acids, biosynthesis of unsaturated fatty acids, plant hormone signal transduction, carbon fixation in photosynthetic organisms, MAPK signaling pathway–plant, carbon metabolism, various types of N-glycan biosynthesis, TCA cycle, and pyruvate metabolism (**Figure 4A**). In contrast, 1,483 downregulated differential genes were enriched in 114 pathways, mainly including some amino acid synthesis and metabolism, starch and sucrose metabolism, galactose metabolism, glycerolipid metabolism, and protein processing and transport (**Figure S4C**). The respiratory metabolism was enhanced, and the substance was synthesized and transported frequently in the seeds at 30 DAR. The 66 differentially upregulated genes were enriched in 38 pathways, mainly including protein processing in the endoplasmic reticulum, starch and sucrose metabolism, circadian rhythm–plant, plant–pathogen interaction, MAPK signaling pathway–plant, plant hormone signal transduction, and synthesis and metabolism of amino acids in the CK_50 and HA_50 groups (**Figure 4B**). The 62 differentially downregulated genes were enriched in 46 pathways, mainly including starch and sucrose metabolism, phenylpropanoid biosynthesis, galactose metabolism, glutathione metabolism, plant hormone signal transduction, and protein processing in the endoplasmic reticulum (**Figure S4D**). These results indicated that plant hormone signal transduction and the MAPK signaling pathway are active during the after-ripening process of *P. notoginseng* seeds.

### Changes in signal transduction in response to exogenous ABA treatment of *P. notoginseng* seeds during after-ripening

**Figure 5:**
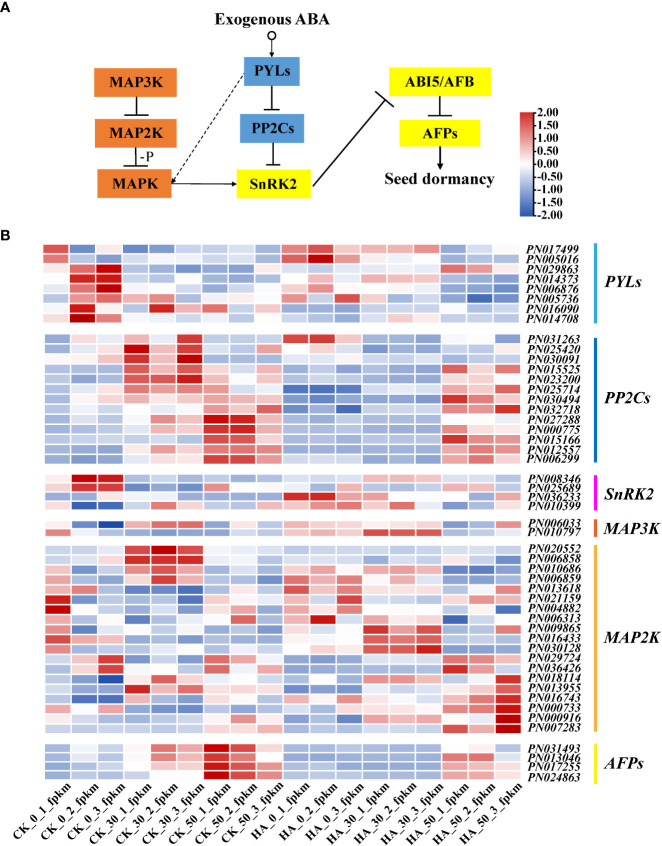
Expression pattern analysis based on RNA-seq of the genes related to the ABA and MAPK signal transduction pathway. **(A)** The pathways of ABA signal transduction and MAPK signaling cascades. Arrows and dotted lines designate directly and indirectly interactions, respectively, and the dashed lines indicate a possible interaction. **(B)** Heat maps of genes associated with ABA signal transduction and MAPK signaling cascades in *P. notoginseng* seeds. In the heat map, different colors indicate the expression level changes.

**Figure 6:**
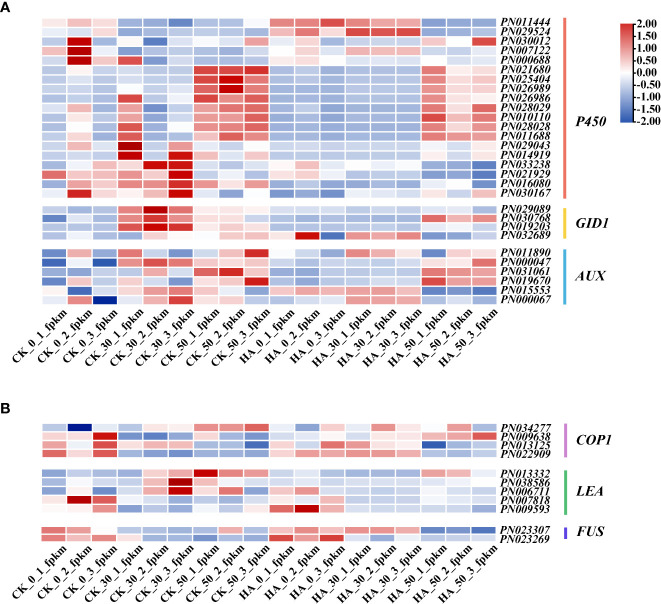
Effects of exogenous ABA on profiles of the transcriptome of *P. notoginseng* seeds during the after-ripening process. **(A)** A heat map showing the expression patterns of the candidate genes involved in GA and IAA signaling responses in *P. notoginseng* seeds. **(B)** A heat map showing the expression patterns of the candidate genes involved in embryonic development in *P. notoginseng* seeds. Different colors indicate the expression level changes.

Some key DEGs of *P. notoginseng* seeds were analyzed, as shown in **Figure 5**. The *PYLs* gradually decreased in the CK during the after-ripening process, and their expressions were downregulated in ABA-treated seeds at 0 DAR and upregulated at 30 DAR and 50 DAR (**Figure 5B**). The *PP2Cs* were gradually increased in the CK, especially at 50 DAR, whereas their expressions were downregulated in the ABA-treated groups (**Figure 5B**). The expression of *SnRK2* was low in the CK but upregulated in the HA-treated groups (**Figure 5B**). The expression of *ABRE-binding transcripts* (*AFPs*) was downregulated in HA-treated seeds (**Figure 5B**). The expression of “*AUX*” (a receptor for the hormone IAA) was downregulated in HA-treated seeds (**Figure 6A**). Additionally, the expressions of *cytochrome P-450 mono-oxygenases* (*P450*) and the *GA* receptor (*GID1*) were significantly suppressed in HA-treated seeds (**Figure 6A**). The *mitogen-activated protein kinase kinase* (*MAP2K*) and *mitogen-activated protein kinase kinase kinase* (*MAP3K*) associated with MAPK signaling cascades were highly expressed in HA-treated seeds (**Figure 5B**). Among the DEGs associated with seed development, *late embryogenesis abundant* (*LEA*) was lowly expressed, while constitutively photomorphogenic1 (*COP1*) and *FUSCA transcription factor* (*FUS*) were relatively active in HA-treated seeds as compared with CK (**Figure 6B**). Therefore, we speculated that exogenous ABA treatment might prolong seed dormancy through the ABA signaling pathway and MAPK signaling cascades during the after-ripening process.

### qRT-PCR validation

**Figure 7:**
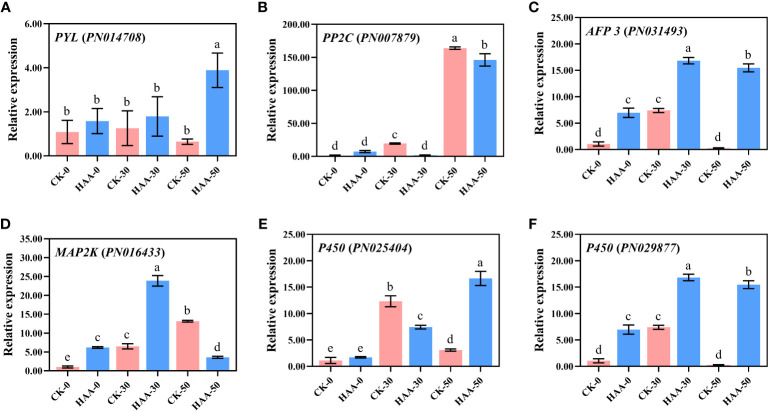
The DEG expression in response to ABA treatment of *P. notoginseng* seeds during the after-ripening process. Gene expressions were analyzed by RT-qPCR. **(A)** *Pyrabactin resistance 1-like* (*PYL*) **(B)** *The protein phosphatase 2C family of genes* (*PP2C*) **(C)** *ABF-binding transcript 3* (*AFP 3*) **(D)** *MAP kinase kinase* (*MAP2K*) **(E)** *Cytochrome P-450 mono-oxygenases* (*P450*) **(F)** *Cytochrome P-450 mono-oxygenases* (*P450*). Values presented are the means ± SE (*n* = 3). Different letters indicate significant differences among treatments in the same period using Duncan’s test (*P <* 0.05).

The candidate DEGs were selected for verification using qRT-PCR (**Figure 7**), and it included genes related to ABA signal transduction (*PYL*, *PP2C*, *AFP*), MAPK signaling cascades (*MAP3K*), and GA Synthesis (*P450*).

## Discussion

### Exogenous ABA promote the dormancy of seed embryos and delay the germination of seeds

The hormone ABA is crucial in regulating seed development and dormancy (Shu et al., 2016b; Brookbank et al., 2021). ABA prevents a seed embryo from continuing to grow when the embryo is completely developed (Raz et al., 2001). Moreover, it has been demonstrated that GAs stimulate the growth potential and weaken the structures surrounding the embryos of *A. thaliana* and tomato (*Solanum lycopersicum*) seeds and thus accelerate the development of seed embryos (Yamaguchi and Kamiya, 2001). In our study, the development of embryos and the germination of seeds were delayed in the ABA-treated groups (**Figure 1**). Similarly, it has been observed that the elongation growth of sprouts is evidently inhibited in the 10.0 mg·l^−1^ ABA-treated *Oryza sativa* seeds (Tang et al., 2003). This indicates that the exogenous hormone ABA might inhibit the elongation growth of the embryo and prevent the space for embryo development from expanding during the after-ripening process.

ABA positively regulates seed dormancy, while GA antagonizes ABA to promote seed germination (Chen et al., 2020; Abley et al., 2021; Zhao et al., 2022). Endogenous ABA is accumulated in *A. thaliana* seeds during the maturation process, which induces and maintains the dormancy of seeds, and then GA content is upregulated to release dormancy during the after-ripening process (Shu et al., 2016b). However, the endogenous ABA was increased and the endogenous GA was decreased in the HA-treated seeds (**Table 1**), and the ratio of ABA to GA was elevated in the ABA-treated seeds of *P. notoginseng* (**Table 2**). We presume that the levels of endogenous hormone and the rate of ABA/GA might be stimulated by exogenous ABA, thus influencing the dormancy of recalcitrant seeds. Further, it has been reported that ABA is accumulated in recalcitrant *Dimocarpus longan* and *Litchi chinensis* seeds during the after-ripening process (Peng and Fu, 1995), and the rate of ABA/GA is increased in dormant *A. thaliana* seeds (Cadman et al., 2006; Shu et al., 2016b; Yang et al., 2022). It has also been demonstrated that germination is inhibited in *A. thaliana* and *T. aestivum* seeds with the exogenous hormone IAA (Ramaih et al., 2003; Zhang et al., 2007). These results indicate that the levels of endogenous hormones are mainly increased by the increased synthesis of endogenous ABA in response to the application of exogenous ABA. Similarly, endogenous IAA was increased under exogenous ABA treatment (**Table 1**), suggesting that increased IAA might enhance seed dormancy. These results are in line with the previous study that found that IAA stimulates ABA signaling to control seed dormancy in *A. thaliana* (Liu et al., 2013b). The JA precursor (12-oxo-phytodienoic acid (OPDA)) cooperates with ABA to inhibit the germination of *A. thaliana* seeds (Varshney and Majee, 2021; Nguyen et al., 2022). Consistently, JA was reduced in the HA-treated seeds than in the CK at 0 DAR (**Table 1**), implying that ABA combined with JA precursor might induce seed dormancy at the early stage of the after-ripening process. Similarly, 80 mg·l^−1^ exogenous ABA treatment delays the germination process of *Chenopodium quinoa* seeds (Wang et al., 2022), as has also been observed in our study (**Figures 1C, D**). These results support the view that ABA treatment could inhibit the development of embryos and delay the germination of recalcitrant seeds.

### The response of endogenous hormone ABA signaling to exogenous ABA in recalcitrant seeds

ABA receptors (PYR/PYL/RCAR family proteins), type 2C protein phosphatase (PP2C) proteins, SnRK2, and *ABA-responsive element binding factors* (*ABF*/*AREB*) respond to ABA and are involved in the regulation of seed dormancy (Fujii and Zhu, 2009; Nakashima et al., 2009; Ali et al., 2021). Similarly, a significant response has been found in the key gene expression of ABA signal transduction including *PYL*, *PP2C*, *SnRK2*, and *ABFs* in HA-treated *P. notoginseng* seeds (**Figure 5**). In the HA-treated seeds, the *PYLs* were upregulated while they were gradually reduced during the after-ripening process (**Figure 5B**). This has also been evidenced by a previous study that found the ABA receptor *PYL* positively regulates the ABA signal transduction pathway in *O. sativa* seeds (Kim et al., 2012). The PYR/PYL/RCAR proteins directly bind to ABA and negatively regulate the protein phosphatase activity of the PP2C protein, which activates the ABA signaling (Wang et al., 2020). Our study showed that the expression of *PP2C*s is lower at 0 DAR and 30 DAR has reversed expression at 50 DAR and is further suppressed under ABA treatment (**Figure 5B**). These findings support the view that members of the PP2C gene family negatively regulates the ABA signaling pathway (Miyazono et al., 2009; Park et al., 2009; Soon et al., 2012). A member of *SnRK2* is also a key positive regulator of ABA signaling (Fujii and Zhu, 2009; Nakashima et al., 2009). *SnRK2s* are released from the inhibition of *PP2Cs* and become active when *PP2C* binds to *ABA* (Fujii and Zhu, 2009; Umezawa et al., 2009; Soon et al., 2012). Then ABA signaling is transmitted *via* the activated kinase SnRK2 phosphorylating various downstream targets, such as the transcription factors *ABF2* and *ABI5* (Miyazono et al., 2009; Kulik et al., 2011). Similarly, the expression of *SnRK2* in CK was high at 0 DAR but almost absent at 30 and 50 DAR, whereas *SnRK2* was consistently active in HA-treated seeds (**Figure 5B**). Unexpectedly, the expression of some *AFPs* was downregulated in HA-treated seeds (**Figure 5B**), suggesting that these transcription factors downstream of ABA might be differentially responsive to the ABA-mediated signaling regulation. In general, our results suggested that the genes (*PYLs*, *PP2Cs*, *SnRK2s*, and *AFPs*) involved in ABA signaling pathway might enhance seed dormancy and consequently delay the germination of *P. notoginseng* seeds with ABA treatment.

The cytochrome P-450 mono-oxygenases catalyze the formation of the GA precursor, GA12-aldehyde, thereby promoting GA synthesis (Pimenta Lange and Lange, 2006). The expression and activity of α-amylase are inhibited in mutant seeds of the receptor for GA signal (*GID1*) resulting in the inability of seeds to germinate (Griffiths et al., 2006). Similarly, it is necessary for seed germination that GA synthesis and GA signal transduction occur in soybean (*Glycine max*) (Shuai et al., 2017). Notably, the expression of *P450* and *GID1* was depressed in the HA-treated seeds, implying that the exogenous hormone ABA might inhibit GA synthesis and signaling transduction, thus suppressing seed germination (**Figure 6A**). The potential involvement of IAA in dormancy maintenance has been recorded, and likewise, IAA receptor mutants dramatically release seed dormancy (Liu et al., 2007). ABA could promote seed dormancy through enhancing IAA signaling in *A. thaliana* seeds (Griffiths et al., 2006). In particular, the expression of *AUX* (the response factor of IAA signaling) was downregulated in HA-treated seeds (**Figure 6A**), implying that IAA shows a diversified response in the regulation network of seed dormancy under ABA treatment. It also indicates that exogenous ABA could delay seed germination by inhibiting the synthesis of endogenous GA and the transduction of GA signals. Thus, we believe that exogenous ABA influences endogenous hormone signals, including the enhancement of the ABA signal and the reduction of the GA signal. This would contribute to promoting seed dormancy and delaying seed germination.

### MAPK signaling cascades strengthen the regulation of embryonic development and seed dormancy with exogenous ABA

The MAPK signaling cascades could affect the phosphorylation of SnRK2 to activate ABA signaling and thus regulate seed germination (Vlad et al., 2009; Hasan et al., 2022). In *O. sativa*, it has been proved that *OsMAPK5* is upregulated by ABA and is involved in ABA signaling transduction (Xiong and Yang, 2003; De Vleesschauwer et al., 2010), and ABA has been shown to regulate *MAP3K* gene expression, including *OsDSM1* (*O. sativa drought-hypersensitive mutant1*) and *OsEDR1* (*O. sativa enhanced disease susceptibility1*) (Kim et al., 2003; Ning et al., 2010). In our study, the expression of *MAP3K* and *MAP2K* associated with the MAPK signaling cascades was upregulated during the after-ripening process (**Figure 5B**). Similarly, *MAPK11* upregulates ABA biosynthesis and phosphorylates SnRK2.2, which then negatively regulates tomato seed germination (Song et al., 2021). Hence, ABA signaling might be amplified through the MAPK signaling cascades to maintain seed dormancy and inhibit seed development. For example, *AtFUS3* influences the development of seed embryos by inhibiting GA biosynthesis and promoting ABA accumulation (Gazzarrini et al., 2004). Consistently, *FUS* was increased in HA-treated seeds during the after-ripening process (**Figure 6B**). The expression of *FUS3* is increased by exogenous ABA in *A. Thaliana*, supporting our results (Kagaya et al., 2005). Thus, we presume that MAPK signaling cascades could amplify the response of some endogenous signals to exogenous ABA and thus regulate the development of seed embryos and prolong seed dormancy. It provides a new method for prolonging the storage of recalcitrant seeds in production by regulating the dormant time.

## Conclusion

**Figure 8:**
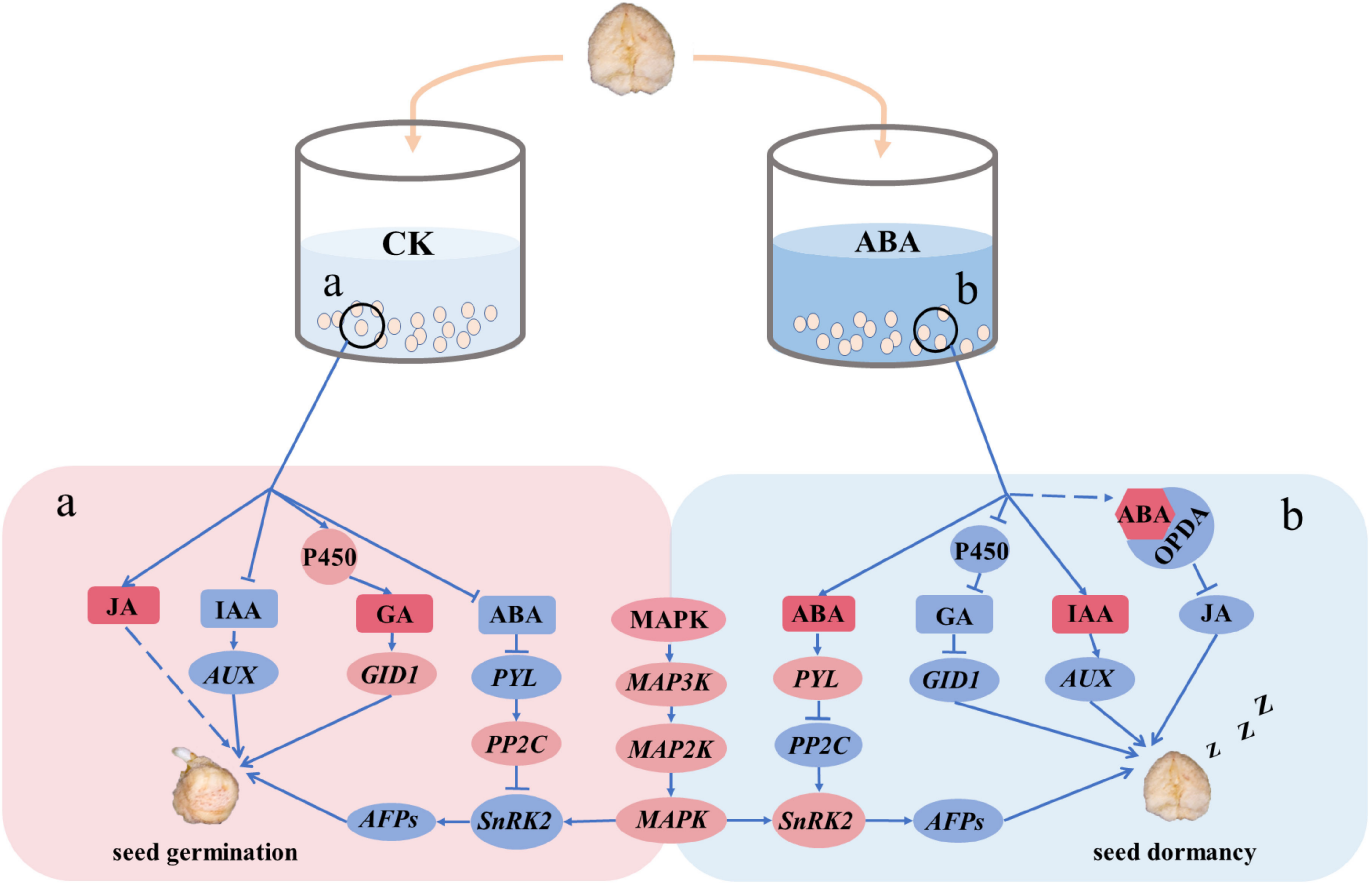
A model explained the possible mechanism of exogenous ABA regulating the dormancy of *P. notoginseng* seeds during the after-ripening process at the transcriptional level. The increased genes are shown in a red circle, and the repressed genes are shown in a blue circle. A red square indicates that the content of hormone is enhanced, and a blue square indicates that the content of hormone is reduced. Arrows and dotted lines designate directly and indirectly interactions, respectively. The dashed lines indicate a possible interaction. (a) The hormonal regulatory network in the CK. (b) The hormonal regulatory network in ABA-treated seeds.

In conclusion, we found that exogenous ABA prolongs dormancy in recalcitrant *P. notogensing* seeds, upregulates ABA signal transduction, and downregulates endogenous GA-synthesis and GA signal transduction. Moreover, MAPK signaling cascades strengthen the regulation of embryonic development and seed dormancy when exogenous ABA is applied during the after-ripening process. In our study, a model is proposed to illustrate the dormant mechanism of recalcitrant seeds in response to exogenous ABA (**Figure 8**). Exogenous hormone ABA could regulate embryonic development, prolong seed dormancy, and delay seed germination of recalcitrant seeds, and MAPK signaling cascades might be involved in amplifying the signaling modulation. These findings provide a novel insight into the dormant regulation of recalcitrant seeds in response to exogenous ABA during the after-ripening process and might contributed to the storage of recalcitrant seeds.

## Data availability statement

The raw sequencing data in this study have been deposited in the Genome Sequence Archive in BIG Data Center (https://bigd.big.ac.cn/), Beijing Institute of Genomics (BIG), Chinese Academy of Sciences, accession number: CRA009677. Other data generated or analyzed during this study are included in this published article and its supplementary information files.

## Author contributions

J-WC provided guidance on the whole process of the experiment and offered suggestions on the writing of the manuscript. Q-YW wrote the manuscript. Q-YW and LY participated most of the experiments. NG, J-SJ, R-MH, CC, Z-GM, and L-GL analyzed the relevant experimental data. All authors listed have made a substantial, direct, and intellectual contribution to the work and approved it for publication.

## Glossary

**Table:**

ABA	abscisic acid
ABF/AREB	ABA‐responsive element binding protein/ABRE‐binding factor
*ABI5*	*ABA insensitive 5*
AFPs	ABI5-binding proteins
BP	biological process
CC	cellular component
CK	the control check
*COP1*	*constitutively photomorphogenic1*
DAR	days of after-ripening process
ddH_2_O	double distilled water
DEGs	differentially expressed genes
Em	embryo
En	endosperm
Em/En	the rate of embryo to endosperm
FPKM	fragments per kilobase of transcript per million fragments mapped
FUS	FUSCA
GA	gibberellin
*GID1*	*GA insensitive dwarf1*
GO	Gene Ontology
HA	10 mg l^−1^ ABA
HPLC-MS	high-performance liquid chromatography-tandem mass spectrometry
IAA	auxin
JA	jasmonic acid
KEGG	Kyoto Encyclopedia of Genes and Genomes
LA	1 mg l^−1^ ABA
*LEA*	*late embryogenesis abundant*
*MAP2K*	*mitogen-activated protein kinase kinase*
*MAP3K*	*mitogen-activated protein kinase kinase kinase*
MAPK	mitogen-activated protein kinase
MF	molecular function
MPD	morphophysiological dormancy
OPDA	12-oxo-phytodienoic acid
*P450*	*P-450 mono-oxygenases*
*PP2Cs*	*type 2C protein phosphatases*
*PYL*	*pyracbactin resistance-like*
*PYR*	*pyracbactin resistance*
qRT-PCR	quantitative real-time PCR
RCARs	regulatory component of ABA receptors
*SnRK2*	*SNF1-related protein kinase subfamily 2*
TCA cycle	citrate cycle
