# Supplementary material for: Exogenous abscisic acid prolongs the dormancy of recalcitrant seed of Panax notoginseng
Source: Front Plant Sci. 2023 Feb 14;14:1054736. doi: 10.3389/fpls.2023.1054736 (PMC9971733; doi:10.3389/fpls.2023.1054736)
Supplement: Supplementary file 1 [file DataSheet_1.pdf]

SUPPLEMENTARY FIGURES

FIGURE S1. Gene expression distribution. The distribution of gene expression levels for different samples is illustrated by box plots. The X-axis represents sample names, the Y-axis on the left indicates the log2 (FPKM + 1). The Box plots for each region are plotted against five statistics (maximum, upper quartile, median, lower quartile and minimum).

FIGURE S2. Pearson correlation analysis of gene expression levels between samples. The X-axis and Y-axis in the graph are the squares of the correlation coefficients for each sample.

FIGURE S3. GO functional annotation of differentially expressed genes (DEGs) in the control and high concentration ABA-treated (HA) *P. notoginseng* seeds during after-ripening process. Top 30 most enriched GO terms of DEGs between CK_0 vs HA_0.

FIGURE S4. KEGG enrichment analysis of differentially expressed genes (DEGs) in the control and HA treated *P. notoginseng* seeds during after-ripening process. (**A**) Top 20 most enriched KEGG pathways of up-regulated genes between CK_0 vs HA_0. (**B**) Top 20 most enriched KEGG pathways of down-regulated genes between CK_0 vs HA_0. (**C**) Top 20 most enriched KEGG pathways of down-regulated genes between CK_30 vs HA_30. (**D**) Top 20 most enriched KEGG pathways of down-regulated genes between CK_50 vs HA_50.

FIGURE S5. KEGG signaling pathway network analysis for plant hormone signaling transduction in the control and HA treated *P. notoginseng* seeds at 30 days after-ripening process. Red indicates up-regulation of gene expression, green indicates down-regulation of gene expression.

FIGURE S6. KEGG signaling pathway network analysis for MAPK signaling pathway-plant in the control and HA treated *P. notoginseng* seeds at 30 days after-ripening process. Red indicates up-regulation of gene expression, green indicates down-regulation of gene expression.

Figure S1

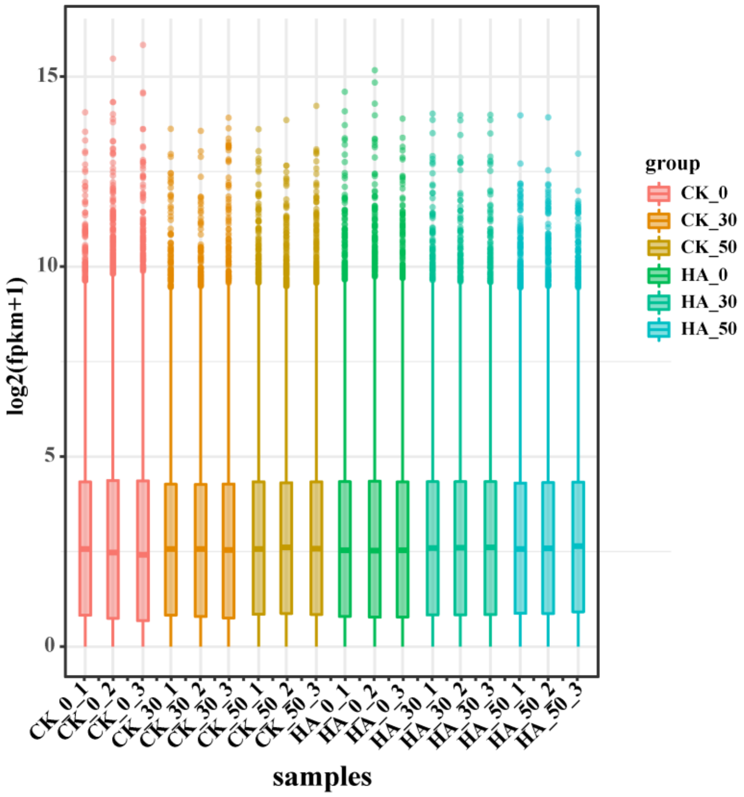

log2(fpkm+1)
15
10
5
0
CK_0_1
CK_0_2
CK_0_3
CK_30_1
CK_30_2
CK_30_3
CK_50_1
CK_50_2
CK_50_3
HA_0_1
HA_0_2
HA_0_3
HA_30_1
HA_30_2
HA_30_3
HA_50_1
HA_50_2
HA_50_3
samples
group
CK_0
CK_30
CK_50
HA_0
HA_30
HA_50

Figure S2

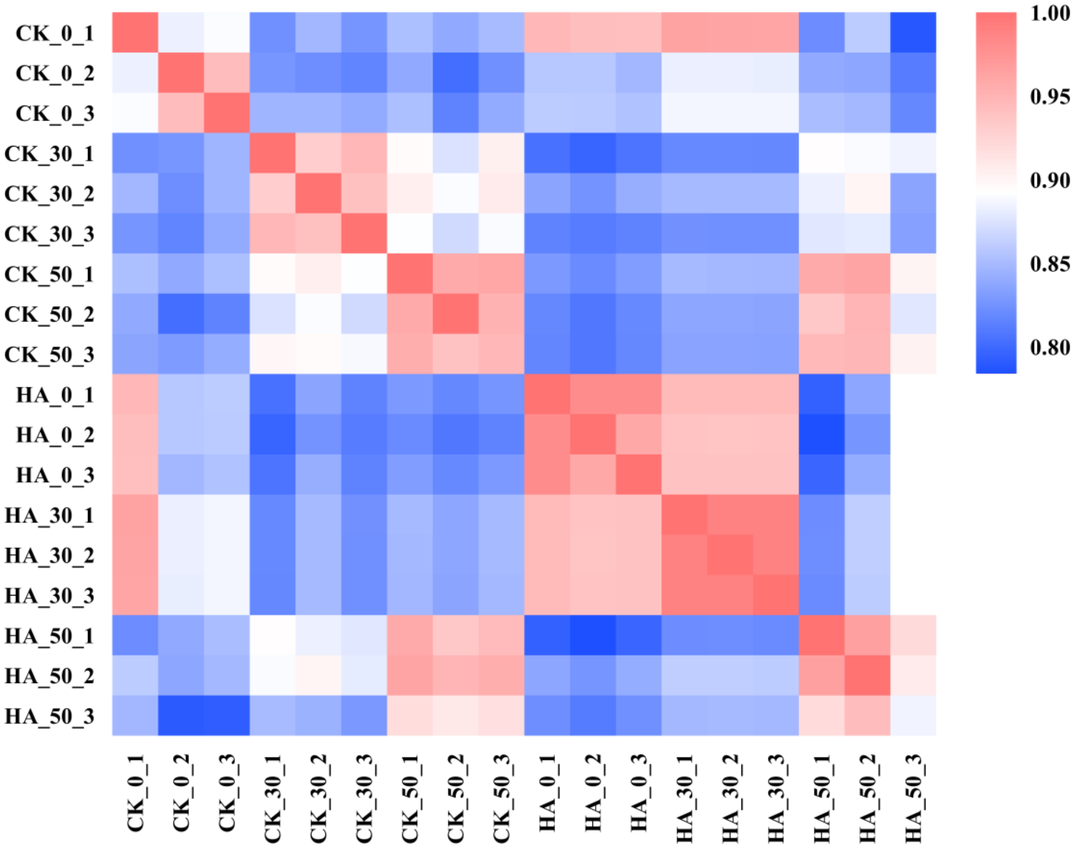
CK_0_1
CK_0_2
CK_0_3
CK_30_1
CK_30_2
CK_30_3
CK_50_1
CK_50_2
CK_50_3
HA_0_1
HA_0_2
HA_0_3
HA_30_1
HA_30_2
HA_30_3
HA_50_1
HA_50_2
HA_50_3
1.00
0.95
0.90
0.85
0.80

Figure S3

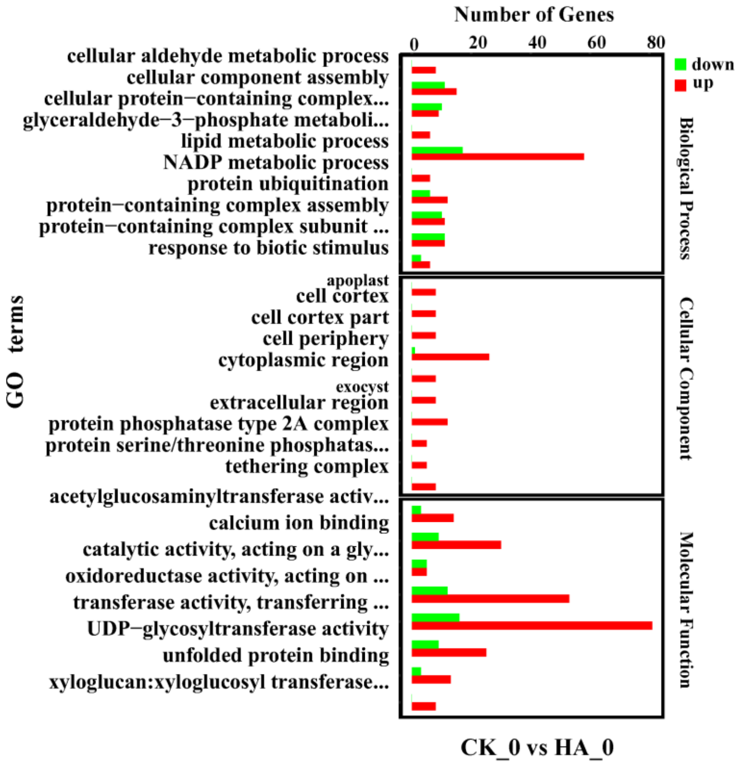

Number of Genes
0
20
40
60
80
down
up
GO terms
cellular aldehyde metabolic process
cellular component assembly
cellular protein−containing complex...
glyceraldehyde−3−phosphate metaboli...
lipid metabolic process
NADP metabolic process
protein ubiquitination
protein−containing complex assembly
protein−containing complex subunit ...
response to biotic stimulus
apoplast
cell cortex
cell cortex part
cell periphery
cytoplasmic region
exocyst
extracellular region
protein phosphatase type 2A complex
protein serine/threonine phosphatas...
tethering complex
acetylglucosaminyltransferase activ...
calcium ion binding
catalytic activity, acting on a gly...
oxidoreductase activity, acting on ...
transferase activity, transferring ...
UDP−glycosyltransferase activity
unfolded protein binding
xyloglucan:xyloglucosyl transferase...
Biological Process
Cellular Component
Molecular Function
CK_0 vs HA_0

Figure S4

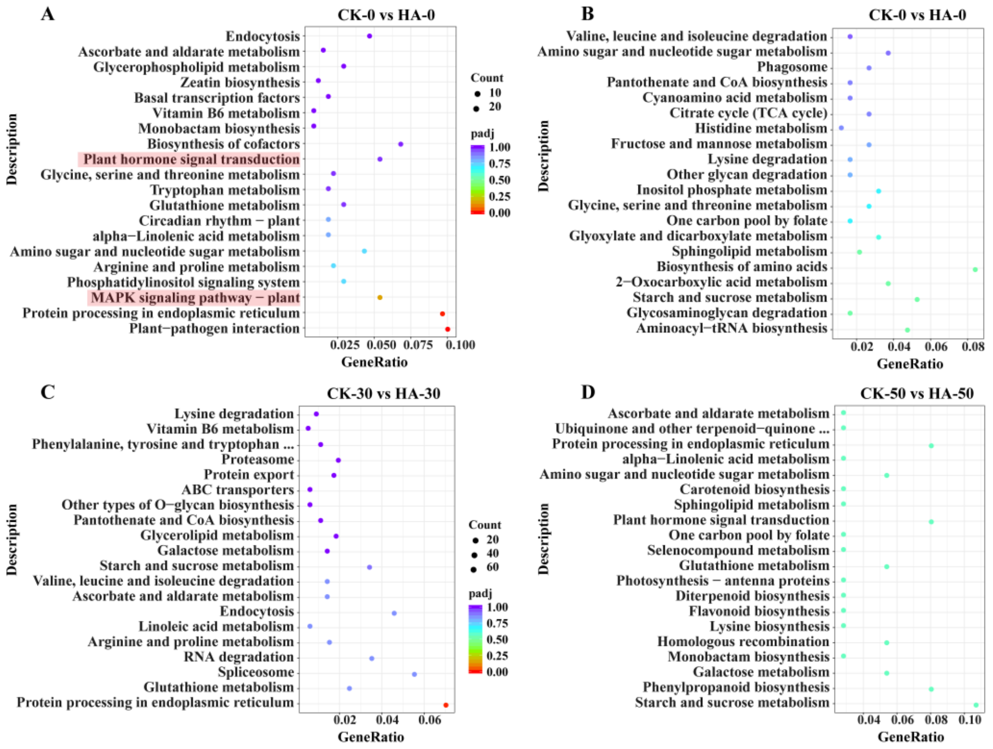

A
CK-0 vs HA-0
Endocytosis
Ascorbate and aldarate metabolism
Glycerophospholipid metabolism
Zeatin biosynthesis
Basal transcription factors
Vitamin B6 metabolism
Monobactam biosynthesis
Biosynthesis of cofactors
Plant hormone signal transduction
Glycine, serine and threonine metabolism
Tryptophan metabolism
Glutathione metabolism
Circadian rhythm − plant
alpha−Linolenic acid metabolism
Amino sugar and nucleotide sugar metabolism
Arginine and proline metabolism
Phosphatidylinositol signaling system
MAPK signaling pathway − plant
Protein processing in endoplasmic reticulum
Plant−pathogen interaction
Description
0.025 0.050 0.075 0.100
GeneRatio
Count
10
20
padj
1.00
0.75
0.50
0.25
0.00
B
CK-0 vs HA-0
Valine, leucine and isoleucine degradation
Amino sugar and nucleotide sugar metabolism
Phagosome
Pantothenate and CoA biosynthesis
Cyanoamino acid metabolism
Citrate cycle (TCA cycle)
Histidine metabolism
Fructose and mannose metabolism
Lysine degradation
Other glycan degradation
Inositol phosphate metabolism
Glycine, serine and threonine metabolism
One carbon pool by folate
Glyoxylate and dicarboxylate metabolism
Sphingolipid metabolism
Biosynthesis of amino acids
2−Oxocarboxylic acid metabolism
Starch and sucrose metabolism
Glycosaminoglycan degradation
Aminoacyl−tRNA biosynthesis
Description
0.02 0.04 0.06 0.08
GeneRatio
C
CK-30 vs HA-30
Lysine degradation
Vitamin B6 metabolism
Phenylalanine, tyrosine and tryptophan ...
Proteasome
Protein export
ABC transporters
Other types of O−glycan biosynthesis
Pantothenate and CoA biosynthesis
Glycerolipid metabolism
Galactose metabolism
Starch and sucrose metabolism
Valine, leucine and isoleucine degradation
Ascorbate and aldarate metabolism
Endocytosis
Linoleic acid metabolism
Arginine and proline metabolism
RNA degradation
Spliceosome
Glutathione metabolism
Protein processing in endoplasmic reticulum
Description
0.02 0.04 0.06
GeneRatio
Count
20
40
60
padj
1.00
0.75
0.50
0.25
0.00
D
CK-50 vs HA-50
Ascorbate and aldarate metabolism
Ubiquinone and other terpenoid−quinone ...
Protein processing in endoplasmic reticulum
alpha−Linolenic acid metabolism
Amino sugar and nucleotide sugar metabolism
Carotenoid biosynthesis
Sphingolipid metabolism
Plant hormone signal transduction
One carbon pool by folate
Selenocompound metabolism
Glutathione metabolism
Photosynthesis − antenna proteins
Diterpenoid biosynthesis
Flavonoid biosynthesis
Lysine biosynthesis
Homologous recombination
Monobactam biosynthesis
Galactose metabolism
Phenylpropanoid biosynthesis
Starch and sucrose metabolism
Description
0.04 0.06 0.08 0.10
GeneRatio

Figure S5

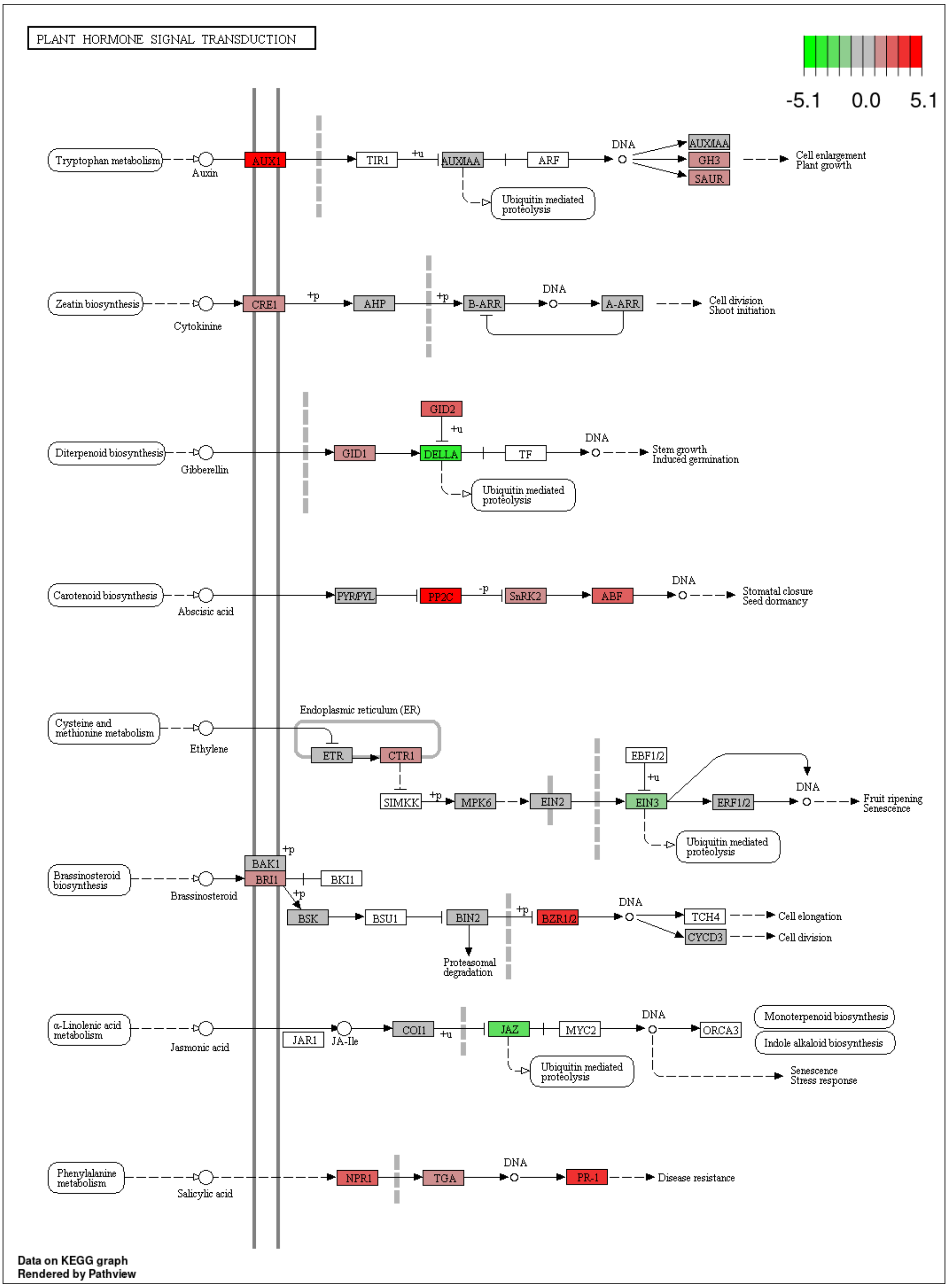

PLANT HORMONE SIGNAL TRANSDUCTION
-5.1 0.0 5.1
Tryptophan metabolism
Auxin
AUX1
TIR1
+u
AUX/IAA
ARF
DNA
AUX/IAA
GH3
SAUR
Cell enlargement
Plant growth
Ubiquitin mediated proteolysis
Zeatin biosynthesis
Cytokinine
CRE1
+p
AHP
+p
B-ARR
DNA
A-ARR
Cell division
Shoot initiation
GID2
+u
Diterpenoid biosynthesis
Gibberellin
GID1
DELLA
TF
DNA
Stem growth
Induced germination
Ubiquitin mediated proteolysis
Carotenoid biosynthesis
Abscisic acid
PYR/PYL
PP2C
-p
SnRK2
ABF
DNA
Stomatal closure
Seed dormancy
Endoplasmic reticulum (ER)
Cysteine and methionine metabolism
Ethylene
ETR
CTR1
EBF1/2
+u
SIMKK
+p
MPK6
EIN2
EIN3
ERF1/2
DNA
Fruit ripening
Senescence
Ubiquitin mediated proteolysis
+p
BAK1
Brassinosteroid biosynthesis
Brassinosteroid
BRI1
BKI1
+p
BSK
BSU1
BIN2
+p
BZR1/2
DNA
TCH4
CYCD3
Cell elongation
Cell division
Proteasomal degradation
α-Linolenic acid metabolism
Jasmonic acid
JAR1
JA-Ile
COI1
+u
JAZ
MYC2
DNA
ORCA3
Monoterpenoid biosynthesis
Indole alkaloid biosynthesis
Ubiquitin mediated proteolysis
Senescence
Stress response
Phenylalanine metabolism
Salicylic acid
NPR1
TGA
DNA
PR-1
Disease resistance
Data on KEGG graph
Rendered by Pathview

Figure S6

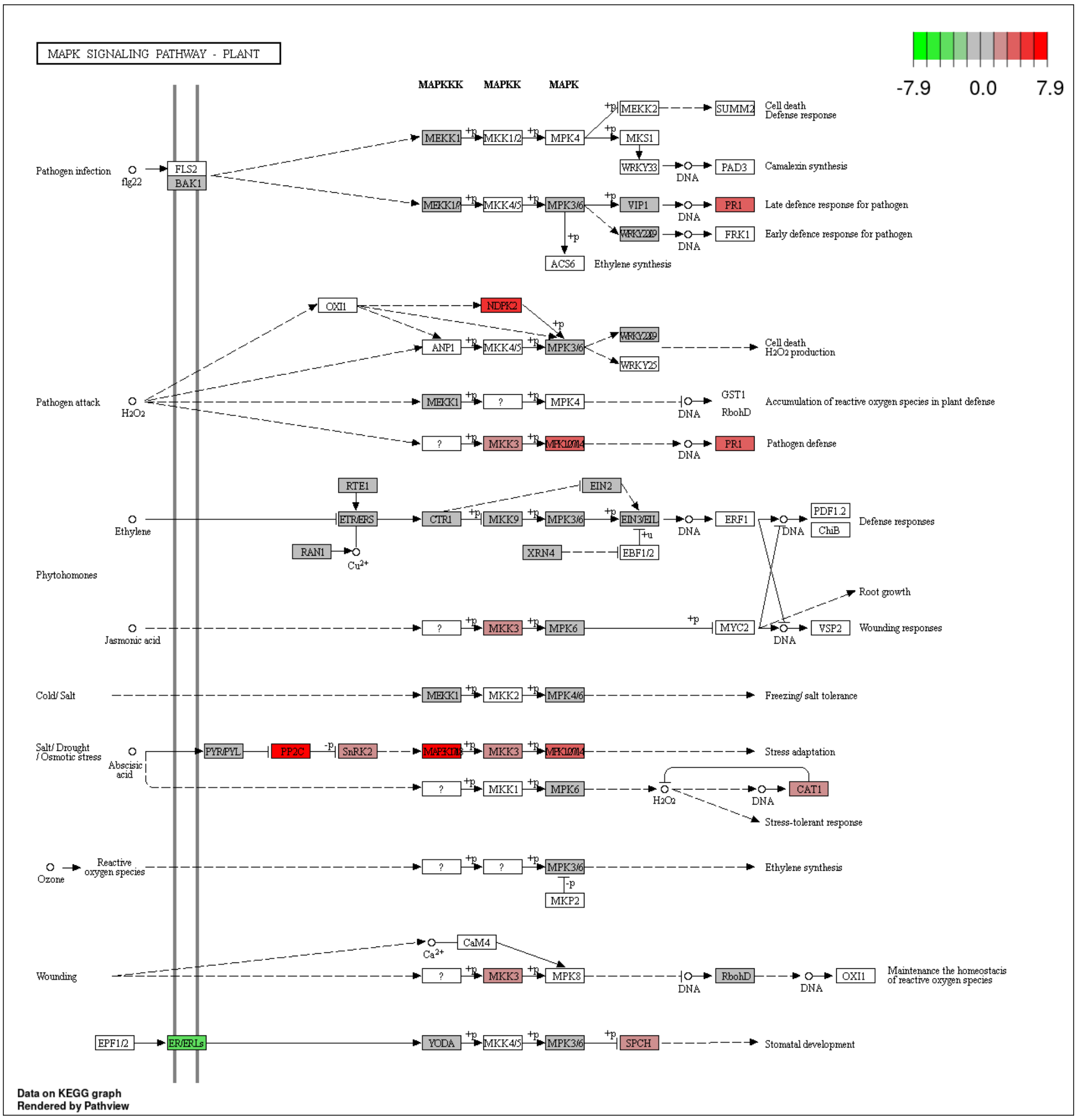
MAPK SIGNALING PATHWAY - PLANT
-7.9
0.0
7.9
MAPKKK
MAPKK
MAPK
Pathogen infection
flg22
FLS2
BAK1
MEKK1
MKK1/2
MPK4
MEKK2
SUMM2
Cell death
Defense response
MKS1
WRKY33
PAD3
Camalexin synthesis
MKK4/5
MPK3/6
VIP1
PR1
Late defence response for pathogen
FRK1
Early defence response for pathogen
ACS6
Ethylene synthesis
OXI1
NDPK2
ANP1
Cell death
H2O2 production
WRKY25
Pathogen attack
H2O2
GST1
RbohD
Accumulation of reactive oxygen species in plant defense
MKK3
Pathogen defense
RTE1
EIN2
Ethylene
ETR/ERS
CTR1
MKK9
EIN3/EIL
ERF1
PDF1.2
ChiB
Defense responses
RAN1
XRN4
EBF1/2
Phytohormones
Root growth
Jasmonic acid
MPK6
MYC2
VSP2
Wounding responses
Cold/ Salt
MKK2
MPK4/6
Freezing/ salt tolerance
Salt/ Drought / Osmotic stress
Abscisic acid
PYR/PYL
PP2C
SnRK2
Stress adaptation
MKK1
CAT1
Stress-tolerant response
Ozone
Reactive oxygen species
MKP2
Ethylene synthesis
CaM4
Wounding
MPK8
OXI1
Maintenance the homeostasis of reactive oxygen species
EPF1/2
ER/ERLs
YODA
SPCH
Stomatal development
DNA
Data on KEGG graph
Rendered by Pathview
