# Supplementary material for: Exogenous abscisic acid prolongs the dormancy of recalcitrant seed of Panax notoginseng
Source: Front Plant Sci. 2023 Feb 14;14:1054736. doi: 10.3389/fpls.2023.1054736 (PMC9971733; doi:10.3389/fpls.2023.1054736)
Supplement: Supplementary file 2 [file DataSheet_2.docx]

# SUPPLEMENTARY TABLES

**TABLE S1 Evaluation of sample sequencing data**

| sample | Raw reads | clean reads | clean bases | error rate  (%) | clean Q20 bases rate  (%) | clean Q30 bases rate  (%) | GC percentage  (%) |
| --- | --- | --- | --- | --- | --- | --- | --- |
| CK_0_1 | 47554084 | 46815702 | 7.02G | 0.02 | 98.02 | 94.08 | 43.71 |
| CK_0_2 | 47956860 | 46919124 | 7.04G | 0.02 | 97.6 | 93.17 | 44.51 |
| CK_0_3 | 47069976 | 45916162 | 6.89G | 0.02 | 97.65 | 93.29 | 44.64 |
| CK_30_1 | 47988046 | 46770276 | 7.02G | 0.02 | 97.73 | 93.39 | 43.79 |
| CK_30_2 | 47121260 | 46087118 | 6.91G | 0.02 | 97.95 | 93.92 | 43.63 |
| CK_30_3 | 52246764 | 50775950 | 7.62G | 0.02 | 97.47 | 92.88 | 44.12 |
| CK_50_1 | 47184604 | 46450880 | 6.97G | 0.02 | 97.87 | 93.8 | 44.1 |
| CK_50_2 | 45327334 | 44037090 | 6.61G | 0.02 | 97.7 | 93.36 | 43.72 |
| CK_50_3 | 50521810 | 49358510 | 7.4G | 0.02 | 97.85 | 93.65 | 44.02 |
| HA_0_1 | 47375378 | 46371594 | 6.96G | 0.02 | 97.41 | 92.63 | 43.71 |
| HA_0_2 | 51135456 | 49958870 | 7.49G | 0.02 | 97.77 | 93.51 | 44.23 |
| HA_0_3 | 46930062 | 46123694 | 6.92G | 0.02 | 97.9 | 93.79 | 43.62 |
| HA_30_1 | 46183192 | 45367036 | 6.81G | 0.02 | 97.72 | 93.36 | 43.76 |
| HA_30_2 | 45523662 | 44814232 | 6.72G | 0.02 | 97.8 | 93.56 | 43.75 |
| HA_30_3 | 47295354 | 45870888 | 6.88G | 0.02 | 97.64 | 93.16 | 43.61 |
| HA_50_1 | 45288052 | 44730048 | 6.71G | 0.02 | 97.52 | 92.97 | 43.8 |
| HA_50_2 | 47251544 | 46684002 | 7.0G | 0.02 | 97.43 | 92.76 | 43.68 |
| HA_50_3 | 47039000 | 45870028 | 6.88G | 0.02 | 97.92 | 93.85 | 43.09 |

**TABLE S2 List of primers used in qRT-PCR analysis**

| Gene ID | Gene name | Primer sequence 5'→3' | | | |
| --- | --- | --- | --- | --- | --- |
|  |  | Forward | Size/bp | Reverse | Size/bp |
| PN014708 | *PYL* | CTGAAGAACACCAAGTAGCGG | 21 | GGCATCTCCAGCAGGATTGT | 20 |
| PN007879 | *PP2C* | ACCACGGGCATTAGTTGAGG | 20 | CTCCACTGGCTGTTACACGT | 20 |
| PN031493 | *AFP 3* | GGGCGATACAAGTGAGAGCA | 22 | CGAAGACCTCACCAGCTTGT | 20 |
| PN016433 | *MAP2K* | ACCACCAACACTTCACCTCG | 20 | CCCAAGGTACACATGCCCAA | 20 |
| PN025404 | *P450* | AGTGCATCGGAAGTGACTGAG | 20 | GTTGCCAAGACTCCTCCTCT | 20 |
| PN029877 | *P450* | GCTGGGCGTATTGTAATGGG | 20 | ACCTCTTCCATCACCAAGGG | 20 |
| PN009353 | *GAPDH* | TGGAATGGCCTTCCGAGTTC | 20 | CGTACCACGCGACAAGTTTC | 20 |

Note: *GLYCERALDEHYDE-3-PHOSPHATE DEHYDROGENASE* (*GADPH*) was used as the reference one. *PYL*: *Pyrabactin resistance 1-like*; *PP2C*: *the protein phosphatase 2C family of genes*; *AFP 3*: *ABF-binding transcript 3*; *MAP2K*: *MAP kinase kinase*; *P450*: *cytochrome P-450 mono-oxygenases*.
